# Benefits of a physician-facing tablet presentation of patient symptom data: comparing paper and electronic formats

**DOI:** 10.1186/1472-6947-13-99

**Published:** 2013-09-02

**Authors:** Daniel Glaser, Sanjula Jain, Philip Kortum

**Affiliations:** 1 Department of Psychology, Rice University, 6100 Main Street, Houston, TX 77005, USA

**Keywords:** Human engineering, Data display, Task performance and analysis, System usability, MDASI

## Abstract

**Background:**

Providing patient information to physicians in usable form is of high importance. Electronic presentation of patient data may have benefits in efficiency and error rate reduction for these physician facing interfaces. Using a cancer symptom measurement tool (the MD Anderson Symptom Inventory (MDASI)) we assessed the usability of patient data in its raw paper form and compared that to presentation on two electronic presentation formats of different sizes.

**Methods:**

In two separate experiments, undergraduates completed two identical six-part questionnaires on two twenty-patient MDASI data sets. In Experiment 1, participants completed one questionnaire using a paper packet and the other questionnaire using an in-house designed iPad application. In Experiment 2, MDASI data was evaluated using an iPad and iPod Touch. Participants assessed the usability of the devices directly after use. In a third experiment, medical professionals evaluated the paper and iPad interfaces in order to validate the findings from Experiment 1.

**Results:**

Participants were faster and more accurate answering questions about patients when using the iPad. The results from the medical professionals were similar. No appreciable accuracy, task time, or usability differences were observed between the iPad and iPod Touch.

**Conclusions:**

Overall, the use of our tablet interface increased the accuracy and speed that users could extract pertinent information from a multiple patient MDASI data set compared to paper. Reducing the size of the interface did not negatively affect accuracy, speed, or usability. Generalization of the results to other physician facing interfaces is discussed.

## Background

Cancer patients often have multiple symptoms and the reporting of these symptoms and their severities provide vital treatment information [1] by determining how well a particular therapy is working either alone or in comparison to other therapies [2]. The development of symptom measurement scales has become the foundation of adequate symptom assessment. One such assessment is the MD Anderson Symptom Inventory (MDASI) which was developed to measure the severity and effect of cancer symptoms [1]. The base MDASI is comprised of 13 core symptoms (e.g., “pain” and “fatigue”) and 6 symptom interference items (e.g., “enjoyment of life”) which assess how symptoms disturb patient function [3]. The MDASI’s combination of a symptom severity scale and a symptom interference scale highlight correlations to condition severity.

Though the MDASI contains pertinent treatment information, it might be difficult for a user of this data to synthesize the data from a large number of different paper sources for multiple patients or multiple visits for a single patient. For example, identifying which items have been most chronic across a patient’s history may be difficult because it requires sorting through multiple MDASI surveys and either recording the information in some idiosyncratic fashion or attempting to hold the information in short-term memory. Further, this kind of task may often be performed in a distracting environment. The goal of this study was to design and assess a physician-facing digital tablet interface that addressed the shortcomings of an MDASI paper presentation. The use of tablet computers and smartphones in the clinical setting has precedent [4-7], with specialized applications already appearing for clinical use [8]. We believe that the comparison of the original form of the MDASI survey with the electronic presentation is an important comparison, since paper tasks may be driven by the original form of the data, as it is collected.

Evaluation was conducted in three parts, starting with a performance comparison between paper and our in-house MDASI tablet interface (Experiment 1), followed by a performance comparison of our tablet interface presented on an iPad and an iPod Touch (Experiment 2). A smaller group of medical professionals participated in Experiment 3 in order to determine if there were differences in user populations. In Experiment 1 the primary aim was to gain insight into how a tablet presentation of MDASI data might affect a user’s ability to quickly and accurately analyze information, not only from a single patient at a point in time (micro level), but also across time and patients (macro level). We were also interested in whether the tablet interface might positively affect a user’s ability to identify symptom/interference trends and locate critical patients from a group. In Experiment 2 we assessed whether reducing the overall size of our tablet interface affects user performance and usability. Experiment 3 was a replication of Experiment 1, using medical professionals as participants.

In summary, this paper is meant to answer three questions: 1) Is a medium-scale electronic presentation (e.g. tablet PC) better than a paper presentation? 2) Can this medium-scale electronic presentation be scaled to smaller, more portable devices (e.g. Smartphones)? and 3) Do these results remain consistent when tested with a target medical professional user population?

### Physician interviews

To gain a greater understanding of how the MDASI was being used in order to optimize a tablet interface, we first interviewed 8 physicians on their use of MDASI data. The interview was designed to gain insight in five areas: the number of patients tracked, the frequency with which physicians are provided MDASI updates from patients, the kinds of information physicians wish to be very prevalent in the presentation, how MDASI information is used along with other medical records to make treatment decisions, and the preferred medium for viewing the information. Each interview was conducted over the phone and lasted approximately 20 minutes.

The number of patients tracked varied greatly, ranging from under 10 to over 700. Physicians indicated that most patients are seen multiple times and that there was a need to track MDASI data over time. Patients completed the MDASI from several times a week to less than once a year. All of the physicians noted that while an item’s criticality is important, of even greater importance is trend information. All of the physicians indicated that symptom information, particularly “Pain”, was more heavily weighted than the MDASI interference items when making treatment decisions and that treatment may be made by assessing individual symptom items rather than looking at the MDASI report as a whole. Physicians noted that it might be valuable to provide numeric thresholds for each item to help identify out-of-range values for the patients. All of the physicians also expressed an interest in being able to track how treatment is affecting scores through some sort of treatment overlay with the trended MDASI score. They were also all open to having the data presented electronically on a tablet or other similarly portable device.

**Figure 1:**
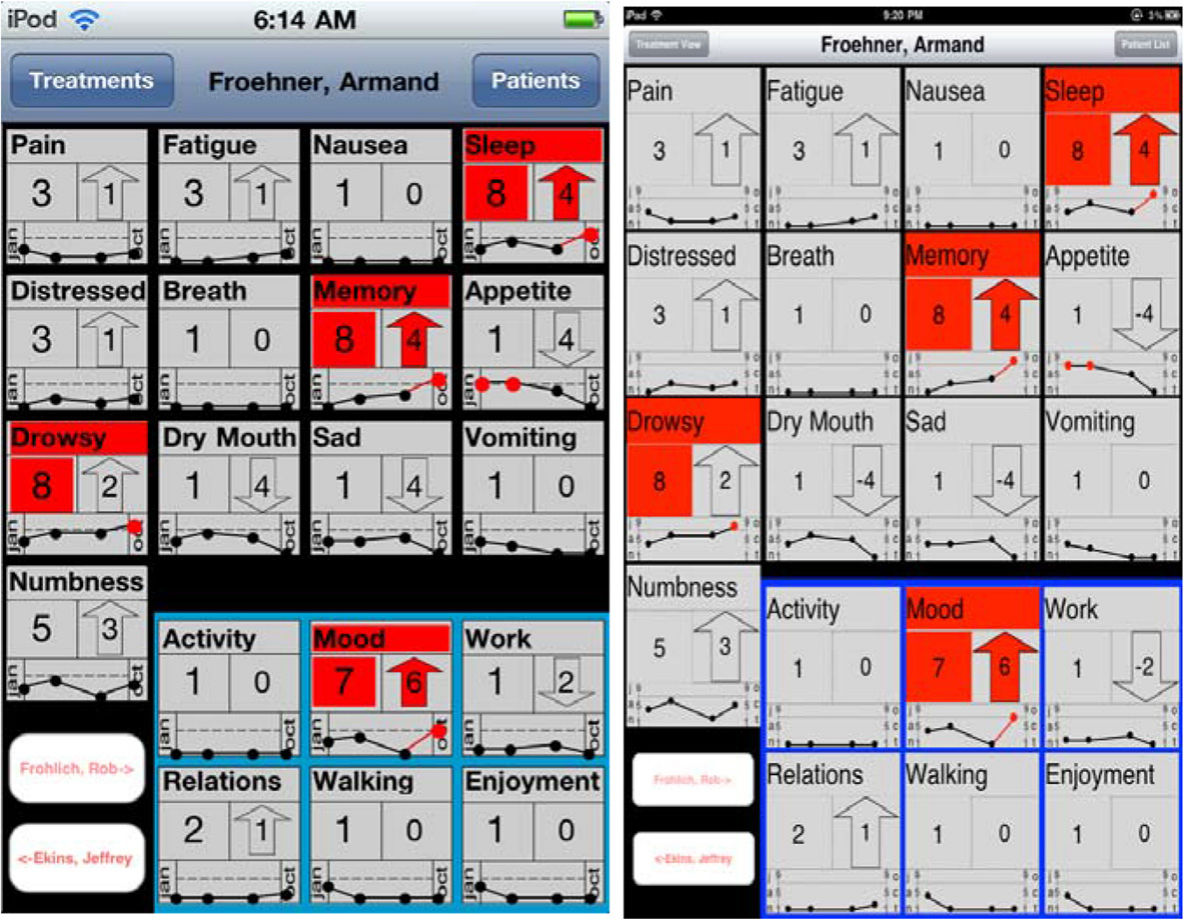
**Electronic interfaces.** The icon view of the electronic MDASI interface as viewed with an iPod (left) on a 3.5 inch diagonal screen and the iPad (right) on a 9.7 inch diagonal screen.

Using this input, a tablet interface was constructed using best practices for the platform [9]. It is known that the design of specific display elements can have an impact on a user’s ability to understand and interpret that data [10,11]. However, it is not enough to present the data in a given format – rather it must have the right visual cues, framed in the right context and be simple enough to be usable [12]. To provide physicians with a snapshot view of a patient’s history across all symptoms, the interface was designed to include an iconic view as shown in Figure 1. The icons serve to provide a holistic view of a given patient’s history, highlight the critical symptoms in color and encapsulate the most recent numerical symptom ratings [13]. Trending information was also included to display a graphical representation of how each symptom rating changed over the past visits [14]. The interface can also be navigated via the patient list feature. The patients are listed in alphabetical order, by last name, and selection of a given patient pulls up the complete MDASI record, in iconic view, for the selected individual. For the purposes of assisting users of the data, patients are prioritized in terms of criticality in this list view using a red color gradient: gray text signaling non-critical patients, and bright red for the most critical [15].

## Method: experiment 1 – paper vs. iPad

Experiment 1 compared the common usability metrics of efficiency and effectiveness [16] of the paper form of the MDASI and the electronic interface as implemented on the iPad across a number of representative tasks. The methodology employed in all of the experiments in this study were in compliance with the Helsinki Declaration and approved by Rice University’s Institutional Review Board Under FWA00003890, IRB0003061.

### Participants

Eighteen undergraduates, drawn from the general student population at Rice University, (10 female, 8 male) participated in Experiment 1. The mean age was 18.9 years (SD = .73). All participants were awarded with experimental credit used in partial fulfillment of classroom requirements.

### Materials

The participants’ primary task was based on the MDASI data of twenty fictitious patients. The patient names were created using a random name generator [17] in order to appropriately model a balanced population considering differences in names based on gender and ethnicity. Each patient’s data set was comprised of four completed MDASI surveys representing the patient’s MDASI six- month history. Patient data was modeled using means and SDs reported by Cleeland, et al. [1]. Patient trends (changes in an item’s value over time) were modeled from a psychometrically validated study that tracked patients’ MDASI item levels of non-small-cell lung cancer receiving chemo-radiation therapy [18]. In our derived patient data set we imagined that the last MDASI data points were from patients in the middle of treatment. Since participants were instructed to answer patient-related data using both the MDASI paper survey and the iPad, two different twenty-patient data sets of similar complexity were prepared. A participant would use one dataset with one presentation format, and then another comparable dataset with the other presentation format. A paper-based data set included twenty manila folders with the patient’s name on the tab stacked in alphabetical order. Each folder included four completed MDASI surveys, each on its own 8.5x11 inch piece of paper, with a timestamp. An iPad-based data set was presented using the iPad application shown in Figure 1. The iPad used was a first generation Apple model MC349LL, which has a 9.7 inch screen with a resolution of 1024x768, running OS 4.3.

A six-question task list was used to gauge how accurately and quickly participants could answer questions about a given set. Taken together, the questions were meant to encompass the kinds of tasks that users of the MDASI might undertake. Three questions asked about information across the entire group of patients, (“How many critical patients do you have?”, “Which patients have x number of critical items?” and “Of the critical patients, what is the single most critical item for each patient?”). These questions are called “Across-Group”. Two questions required users to assess a single patient’s history (“Name the symptoms that have gotten worse since patient x’s last visit” and “For patient x, which symptom has been at or above the critical threshold throughout the recording period?”). These questions are called “Across-Patient”. One question inquired about information from only a single MDASI survey for a single patient (“For patient x, indicate the 3 most critical symptoms”). This question is called “Single-Survey”. Finally, the System Usability Scale (SUS) [19] was used to measure the overall usability of the iPad application. The SUS is a 10 item questionnaire that produces scores in the range of 0–100, where higher scores indicate better usability.

### Procedure

After signing an IRB-approved consent form, participants were given a brief description of the MDASI and provided a tutorial where they were shown the various features of the interface including important interface elements and trained how to navigate between the icon and trend screen views. Participants were also instructed that red indicated a critical item and that criticality was defined as any symptom item that earned a score of 7 or higher on the last visit or had an increase of 4 or more from the third to last visit. A critical patient was defined as any patient that had at least one critical item. Next, depending on which would be used first, participants were given instruction on either the paper packet or iPad application. Participants were then given the questionnaire and instructed to answer each question as quickly as possible while remaining accurate. At the start of each question, participants were asked whether they understood the task. If they indicated that they understood, the experimenter would start the timer and the participant would begin examining the medical records to form a response. The timer was stopped when the participant indicated that the answer to the question was complete. After all items were answered, participants were given instruction on the alternative method and then completed the same six questions on a different data set. Both the data presentation mode (paper or iPad) and data set order were counterbalanced. Participants were assigned, in arrival order, to one of 4 data conditions; Paper first using data set 1 followed by iPad using data set 2, Paper first using data set 2 followed by iPad using data set 1, iPad first using data set 1 followed by Paper using data set2, or iPad first using data set 2 followed by Paper using data set 1. After using the iPad, participants were asked to complete the SUS.

### Questionnaire scoring

Questionnaire accuracy was assessed on a question-by-question basis. For all but two questions, a score of one was assigned if the answer was correct and zero if not correct. The remaining two questions had multiple parts: the question “Which patients have x number of critical items?” had 5 parts, for x = 1-5 and the question “Of the critical patients, what is the single most critical item for that patient?” had 5 parts as well. The accuracy for these multi-part questions was calculated as (1/number of parts * number of correctly answered parts). A participants’ overall questionnaire accuracy was the average accuracy of the six questions.

## Experiment 1 Results

### Accuracy

Using a Wilcoxon Signed Rank test, we found that participants were more accurate when using the iPad (Mean (M) = .79, Standard Deviation (SD) = .20, Median (Mdn) = .80, Interquartile Range (IQR) = .31) than they were with paper (M = .58, SD = .17, Mdn = .63, IQR = .23), *Z* = 3.1, *p* = .0019. Since Across-Group or Across-Patient questions require information from multiple MDASI surveys, it was expected that these items would have a particularly high iPad advantage. Using a Wilcoxon Signed Rank test we found an iPad advantage for only Across-Group items (*Z* = 3.56, *p* = .0004). No significant advantage was found for the Single-Survey group or Across-Patient group.

### Task completion time

Using a Wilcoxon Signed Rank test, we found that participants were significantly faster (>4x) when using the iPad (M = 94 s, SD = 15.7 s, Mdn = 98.5 s, IQR = 25.8 s) than when using paper packets (M = 394 s, SD = 122.8 s, Mdn = 372 s, IQR = 170.3 s), *Z* = 3.71, *p* = .0002. Unlike the accuracy measure, a significant response speed advantage for the iPad was observed for each of the question types. Using a Wilcoxon Signed Rank Test we found significant differences for Across-Group (*Z* = 3.71, *p* = .0002), Across-Patient (*Z* = 3.71, *p* = .0002) and Single-Survey (*Z* = 2.3, *p* = .0214).

### Usability

The iPad’s average SUS score was 91/100 which is within the ‘excellent’ range [20].

## Method: experiment 2 – iPad vs. iPod

One potential problem with the iPad in a clinical context is its relative lack of portability compared to smartphones, making it difficult for physicians to keep it on their person. Modern smartphones, which are as functional as tablets, may be a potential solution to this portability problem. A cause for concern, however, is that the smaller smartphone screen may negatively impact readability, since interface elements will be restricted to a much smaller area. To examine these issues, Experiment 2 compared the efficiency, effectiveness and satisfaction of the iPad interface of the MDASI and an equivalent version presented on an iPod Touch interface.

### Participants

Forty undergraduates, drawn from the general student population, (25 female: 15 male) participated in Experiment 2. The mean age was 19.1 years (SD = 1.15). As in Experiment 1, all participants were awarded with experimental credit that was used in partial fulfillment of class requirements.

### Materials

The same Experiment 1 six-question questionnaire, data sets, and usability assessment (SUS) were used to assess data performance and usability. As with the paper, the iPod prototype had two data sets loaded on it, allowing the device and data set order to be properly counterbalanced. An iPod, model MC086LL, which has a 3.5 inch display with a resolution of 320x480, running iOS 4.0 was used in the experiment, as it has a nearly identical form to common smartphones, most notably the iPhone, but was easier to administer for the experiment.

### Procedure

The same design and procedure was used in Experiment 2 as in Experiment 1 with the exception that both mediums were assessed with the SUS after being used.

## Experiment 2 Results

### Accuracy

Questionnaires were scored in the same fashion as in Experiment 1. Using a Wilcoxon Signed Rank test, we found that there was no difference in the accuracy of iPad responses (M = .84, SD = .18, Mdn = .83, IQR = .33) and the iPod (M = .80, SD = .19, Mdn = .83, IQR = .33), *Z* = 1.09. *p* = .2757. None of the question type groupings were found to be answered more accurately when using the iPad.

### Task completion time

Using a Wilcoxon Signed Rank test, we found that there was no difference in task completion time between the iPad (M = 89.9 s SD = 34.4 s, Mdn, 82.2 s, IQR = 33.9 s) and the iPod (M = 84.8 s, SD = 19.8 s, Mdn = 80.7 s, IQR = 14.6 s), *Z* = .22, *p* = .8259. No significant response speed advantages were observed for any question type.

### Usability

Using a Wilcoxon Signed Rank test, we found that there was no difference in the average SUS scores for the iPad (M = 81.8, SD = 11.8, Mdn = 85.0, IQR = 15.6) and iPod (M = 78.3, SD = 13.0, Mdn = 80.0, IQR = 20.0). *Z* = 1.63, *p* = .1031.

## Method: experiment 3 – paper vs. iPad for medical professionals

### Participants

Ten medical professionals (5 female, 5 male) participated in Experiment 3. Eight of these participants were physicians and two were registered nurses. The mean age was 45.4 years (SD = 9.9). All participants were offered a gift card for their completion of the study, although some declined this incentive.

### Materials

The exact materials used in Experiment 1 were used in Experiments 3, except that only 4 questions were used for brevity. One Across-Group and one Across Patient question was removed from the question set.

### Procedure

The exact procedure used in Experiment 1 was used to conduct Experiment 3, with the exception that both mediums were assessed with the SUS after being used.

## Experiment 3 Results

### Accuracy

Using a Wilcoxon Signed Rank test, we found that participants were more accurate when using the iPad (M = .61, SD = .26, Mdn = .50, IQR = .25) than they were with paper (M = .23, SD = .25, Mdn = .25, IQR = .25), *Z* = 2.78, *p* = .0054. Using a Wilcoxon Signed Rank test we found an iPad advantage for only Across-Group items (*p* < .01). No significant advantage was found for the Single-Survey group or Across-Patient group, mirroring the results found in Experiment 1.

### Task completion time

Using a Wilcoxon Signed Rank test, we found that participants were faster when using the iPad (M = .39.3 s, SD = 19.1, Mdn =43.1, IQR = 17.1) than they were with paper (M = 246.3, SD =103.4, Mdn = 275.4, IQR = 130.8), *Z* = 2.78, *p* = .0054. Using a Wilcoxon Signed Rank Test we found significant differences in task completion times for Across-Group (*Z* = 2.78, *p* = .0054), Across-Patient (*Z* = 2.78, *p* = .0054) and Single-Survey (*Z* = 2.68, *p* = .0074) questions, again mirroring the results for Experiment 1.

### Usability

Using a Wilcoxon Signed Rank test, we found that the average SUS score was significantly higher for the iPad (M = 85.0, SD =19.8, Mdn =93.8, IQR =25.6) than for paper (M = 58.8, SD = 20.0, Mdn = 53.8, IQR = 14.4), *Z* = 2.37, *p* = .0178.

## Discussion

The results of this research demonstrate that the electronic form of the data is significantly more effective in terms of supporting accurate and fast data examination, both of which have high importance when medical professionals are trying to use the information to make treatment decisions. This increase in accuracy and speed is likely due to the fact that when the data is presented in its paper format, physicians are faced with a large array of data but have no systematic way of comparing multiple time points or patients. Data from the medical professionals participants (Experiment 3) showed the same trends as the data from the student group (Experiment 1). These findings are different than those reported by Krauskopf and Ferrell [21], who found only improvements in efficiency for medical professionals using a digital tablet to gather information.

Though there was an overall advantage both in accuracy and speed, it is important to note that only the Across-Group questions were answered significantly more accurately on the iPad, whereas all questions were answered significantly more quickly using the iPad. Though Single-Subject and Across-Patient questions did not appear to be particularly error prone, being able to identify this information more quickly in a clinical setting would perhaps give medical professionals more time to interact face-to-face with patients.

The data for the paper vs. iPad comparison indicate a minimal difference in the accuracy of the participants for this set of tasks. Given that micro-level questions are specific to certain patients, and even more so, specific classes of survey items, there was less information that participants needed to integrate and analyze in order to answer the questions. Processing larger amounts of information would pose a greater cognitive workload and would prove to be a more difficult task, inevitably leaving space for inaccuracies. At the macro level, there is a significant difference in the accuracy for two Across-Group questions in which participants scored much higher using the iPad.

Perhaps the most surprising finding was that Across-Patient questions were not answered more accurately using the iPad. There are three factors that likely contributed to this non-differentiation. First, it may have been that participants occasionally misunderstood the question. Often, participants would ask whether the numerical rating took precedence over the rating change between visits in determining which item(s) were most critical. This is an important consideration because some patient data showed trends in which there was a significant negative change in a patient’s condition in two consecutive earlier visits presented with the most recent visit’s symptom rating reported as non-critical. However, the guidelines given prior to the experiment indicated that a critical patient considered negative changes between the third and fourth visits only. It was observed that some questionnaire responses were swayed by the significant early patient history, which contradicts the criteria given for accurate evaluation. Uncertainty regarding these factors may have unduly influenced the participant’s judgments. These misunderstandings would lead to accuracy rates that would not be affected by the data medium, diluting the potential iPad advantage. We predict that these mistakes will be less frequent in a clinical setting where the user will self-define the task. Second, Across-Group questions required the synthesis of 20 MDASI surveys, while Across-Patient questions required synthesizing MDASI information from just 4 surveys. Though tracking a four-point patient history is relatively time consuming using paper, it is still cognitively tractable [22]. Of note, however, is the possibility that the mini-trend view (presented in the icon view, as shown in Figure 1) might be misread due to its inherent size limitations. The expanded view mode helps mitigate this possibility, but further improvements to the small representation are being explored. Finally, it is quite likely that some participants did not fully appreciate the difference between symptom and interference items. Even though the participants were instructed that the MDASI has two types of items, we cannot ensure that this information was remembered at critical moments. This seems plausible considering the analysis of incorrect responses which showed answers that accounted for items in both categories as opposed to making a distinction. For those that are unfamiliar with the MDASI (our participants for example), the paper labeling may have been particularly helpful. On the iPad the two types of items were not labeled but instead separated by background color and location. We expect, however, that those using the iPad interface in the clinical setting will have enough knowledge of the MDASI to make labeling unnecessary. Future studies in a clinical setting could verify this supposition.

### Usability evaluation

The SUS scores provide an easy and robust way to evaluate the usability of a wide range of interfaces [20], including the MDASI. An average SUS score above 70 is deemed acceptable and is the target range for the MDASI interface [20]. The SUS scores in the paper vs. iPad evaluation are comprised of iPad ratings all above 76. Thus, the iPad earned a very high usability score, with a large concentration of scores in the 90s range, corresponding to adjective ratings of ‘good-excellent’ [23].

Without a doubt, the paper interface was the most difficult to use. Answering all 6 of the questions using the paper folders took about an hour on average to complete. In order to answer the questions, the participants had to manually make notes and work to see trends and make comparisons. The iPad interface was a sharp contrast to the paper task, in that no manual work was involved. The iPad task was focused on interpretation of data that was already organized in a strategic manner.

### Screen size

Using a smartphone to view medical records is advantageous because the smartphone is quite portable. Physicians can slip the device in their pocket and walk around unencumbered while retaining the ability to view medical records both whenever and wherever they happen to be. A potential disadvantage of a smartphone, however, is its relatively small screen size compared to other connected devices such as desktops, laptops, and more recently iPad-like tablets. When transposing information to a smaller screen, as we did with our tablet interface, information concessions are necessary. Either the designer will be forced to present less information on the screen or decrease the size of the information (e.g., reduce text and figure sizes). For our iPod prototype, we made the latter concession. In our comparison of the iPad and iPod, even though information elements were dramatically reduced in size on the iPod, we did not find a significant decrease in usability ratings. Gutwin and Feda [24] in their investigation of performance differences between large and small screens found that navigation methods such as panning were ineffective on small screen devices compared to techniques such as fisheye view and two-level zooming. In the MDASI interfaces, we were careful to ensure that the visual elements of the larger iPad interface were visible on the iPod interface without the need for additional zooming. The navigation scheme was the same on both devices with the exception of the patient list, which required scrolling at times on the iPod because not all 20 patients name could fit on the screen. This need to scroll was one of the biggest complaints of the iPod, indicating that if the interface was designed in a fashion such that scrolling was often necessary in many parts of the interface, usability ratings likely would not have been matched. Clearly the iPad interface was conservative in its use of space, and more information could be presented on the main screen if necessary – however, this expansion of information on the iPad would make direct transfer of the interface to smaller screens problematic. We believe that consistency of interfaces to the greatest degree possible across delivery platforms is important, so the conservative design of the larger screen is warranted.

## Conclusions

We evaluated common metrics of usability (effectiveness, efficiency and satisfaction) on three different MDASI data presentation mediums: paper, iPad, and iPod Touch. The results of the study indicate that presenting this information using electronic formats can increase the effectiveness, efficiency and satisfaction of the person using the data when compared to using and interpreting data in it original paper form. Moreover, the split in effectiveness, efficiency, and satisfaction occurred between paper and the digital mediums; screen size did not have a significant effect on these metrics. These results suggest that well designed interfaces for the presentation of patient information can help decrease the amount of time that is required to assess a patient’s condition while simultaneously reducing the number of errors made in medical assessments. Further research in clinical setting should be explored to validate these findings.

## Abbreviations

MDASI: MD Anderson symptom inventory; SUS: System usability scale.

## Competing interests

The authors declare they have no competing interests.

## Authors’ contributions

DG coded the electronic interfaces and led the statistical analysis of the data and assisted in the student data collection effort. SJ led the data collection effort for students and physicians. PK generated the experimental design and created the design of the electronic interfaces with DG. All authors participated in the writing of the paper. All authors read and approved the final manuscript.

## Pre-publication history

The pre-publication history for this paper can be accessed here:

http://www.biomedcentral.com/1472-6947/13/99/prepub
